# Impact of renal impairment on pharmacokinetics, safety, and tolerability of funobactam, combined with imipenem and cilastatin

**DOI:** 10.1128/aac.00808-26

**Published:** 2026-08-27

**Authors:** Keith A. Rodvold, Grigor Mamikonyan, Yu Wang, Anastasia McRoberts, Dyal Garg, Richard Preston, Thomas Marabury, Meijie Le, Jianguo Li

**Affiliations:** 1 University of Illinois Chicago 14681, Chicago, Illinois, USA; 2 Clinartis LLC, Hollywood, Florida, USA; 3 Evopoint Biosciences Co Ltd 662696 , Suzhou, China; 4 Pharmaron Germantown, Germantown, Maryland, USA; 5 Clinical Research Services Inc., Boynton Beach, Florida, USA; 6 Division of Clinical Pharmacology, University of Miami, Miami, Florida, USA; 7 Orlando Clinical Research Center, Orlando, Florida, USA; 8 Evopoint Biosceinces USA, Inc., Concord, Massachusetts, USA; University of Houston, Houston, Texas, USA

**Keywords:** safety, renal impairment, pharmacokinetics, cilastatin, imipenem, XNW4107, funobactam

## Abstract

A phase 1, open-label study was conducted to investigate the effect of renal impairment (RI) on pharmacokinetics (PK) and safety of funobactam plus imipenem/cilastatin. Participants were enrolled and assigned to one of five cohorts based on renal function category: normal renal function, mild RI, moderate RI, severe RI, and end-stage renal disease (ESRD) requiring hemodialysis (HD). Participants received a single-dose of funobactam with imipenem and cilastatin by intravenous infusion administered over 60 min. Serial blood and urine samples were collected for non-compartmental PK analysis. Compared to participants with normal renal function, the geometric mean ratio (90% confidence interval) of plasma area under the curve (AUC0-∞) for funobactam, imipenem, and cilastatin increased with RI from mild (1.32 [1.04, 1.66], 1.37 [1.04, 1.79], and 1.27 [0.90, 1.78], respectively) to ESRD requiring HD (9.58 [7.59, 12.10], 2.69 [2.06, 3.52], and 15.15 [10.8, 21.3], respectively). The renal clearance for funobactam, imipenem, and cilastatin decreased progressively from normal renal function (3.93 ± 1.12 L/h, 4.95 ± 2.40 L/h, and 6.98 ± 2.75 L/h, respectively) to severe RI (0.94 ± 0.37 L/h, 1.16 ± 0.34 L/h, and 1.70 ± 0.60 L/h, respectively). Plasma clearance of funobactam, imipenem, and cilastatin correlated (r ≥0.93) with creatinine clearance and the estimated glomerular filtration rate. The treatment was well tolerated, with no deaths, severe adverse events, or discontinuation due to adverse events. Dosage adjustments of funobactam, imipenem, and cilastatin will be needed in patients with renal impairment or ESRD requiring HD.

**CLINICAL TRIALS:**

This study is registered with ClinicalTrials.gov as NCT04787562.

## INTRODUCTION

Funobactam (formerly XNW4107) is a potent selective inhibitor of Ambler Classes A, C, and D β-lactamase and, when combined with imipenem, has exhibited significant antimicrobial activity against resistant pathogens, including carbapenem-resistant *Enterobacterales* (CRE), carbapenem-resistant *Acinetobacter baumannii* (CRAB), and carbapenem-resistant *Pseudomonas aeruginosa* (CRPA) in a series of *in vitro* and *in vivo* studies (1–3). Funobactam is being developed for the treatment of serious infections with unmet medical need, including, but not limited to, complicated urinary tract infections, complicated intra-abdominal infections, and nosocomial pneumonia, including ventilator-acquired bacterial pneumonia, caused by gram-negative pathogens, including CRE, CRAB, and CRPA.

Funobactam has demonstrated excellent linear and dose-proportional PK properties during single ascending dose (SAD) and multiple ascending dose (MAD) studies with a single dose of 50–1,250 mg, and multiple repeated doses of 167–500 mg, given every 6 h (q6h) for 7 days (4). At the funobactam dose of 250 mg, the estimated mean total body clearance (CL), volume of distribution at steady-state (V_SS_), and elimination half-life (t_1/2_) following intravenous dose administration were 5.71 L/h, 17.5 L, and 3.0 h, respectively. The average urinary recovery of funobactam across the different dose groups ranged from 80% to 100%. Imipenem and cilastatin are eliminated primarily by the kidney (up to approximately 70% following the single-dose administration of imipenem/cilastatin) with a mean t_1/2_ of 1 h (5–7). There was 30%–40% accumulation for funobactam after multiple repeated q6h dose administration, while there was no accumulation for imipenem/cilastatin after multiple repeated dose administrations. In an exploratory drug-drug interaction study, no clinically meaningful changes in the mean exposure ratios were observed between funobactam and imipenem/cilastatin administered alone or in combination (4). In addition, *in vitro* studies showed that funobactam is not a substrate and unlikely to be a potential inhibitor of major transporters, including P-gp, BCRP, OATP1B1, OATP1B3, OAT1, OAT3, OCT2, MATE 1, and MATE2-K. A good safety profile with funobactam was observed when administered alone or in combination with imipenem/cilastatin as a single dose or multiple repeated doses.

Funobactam has demonstrated a slightly higher penetration into the pulmonary epithelial lining fluid (ELF) compared to imipenem (8). The estimated ratios of ELF to unbound plasma concentrations based on area under the plasma concentration time curves (AUC) from time 0 to 6 h (AUC_0–6h_) values for funobactam and imipenem were 35.2% and 31.8%, respectively. Funobactam penetrated alveolar macrophages (AM) and exhibited more than twice the intrapulmonary penetration than imipenem. The estimated AUC_0–6h_ ratio of AM to unbound plasma for funobactam and imipenem was 36.6% and 16.8%, respectively.

The primary objective of this study was to evaluate the pharmacokinetics of funobactam, imipenem, and cilastatin after single-dose concurrent intravenous administration in participants with normal renal function, participants with various degrees of renal insufficiency, and participants with end-stage renal disease (ESRD) requiring intermittent hemodialysis (HD). The secondary objective of this study was to assess the safety and tolerability of funobactam, imipenem, and cilastatin after single-dose concurrent intravenous administration in participants with various degrees of renal function (ClinicalTrials registration no. NCT04787562).

## RESULTS

Thirty-nine adult male and female participants were enrolled and assigned to one of five cohorts. The study completed planned enrollment (eight participants per cohort) for Cohorts 1 (normal renal function), 2 (mild RI), 3 (moderate RI), and 5 (ESRD requiring HD). Cohort 4 (severe RI) enrolled seven participants, and no further enrollment for this cohort was conducted since the minimum required number of evaluable participants (*N* = 6) per protocol was met. Demographic and baseline characteristics were similar across cohorts, with the exception of race and ethnicity in participants with ESRD requiring HD (Table 1). Overall, the study population was predominantly male (74.4%) with a mean (standard deviation [SD]) age of 63.5 (11.5) years and body mass index (BMI) of 29.5 (2.79) kg/m^2^. The majority of participants in Cohorts 1–4 were White, whereas 7 of 8 participants in Cohort 5 (ESRD requiring HD) were Black. Baseline renal function as estimated glomerular filtration rate (eGFR) ranged from 4 to 110 mL/min/1.73 m^2^ and calculated creatinine clearance (CL_CR_) ranged from 7 to 128 mL/min (9, 10).

**TABLE 1:** Demographic and baseline characteristics^*a*^

Demographic or characteristic	Category or statistic	Normal renal function (*N* = 8)	Renal impairment				Overall (*N* = 39)
			Mild (*N* = 8)	Moderate (*N* = 8)	Severe (*N* = 7)	ESRD (*N* = 8)	
Age (years)	Mean (SD)	63.9 (3.80)	66.9 (6.81)	70.4 (9.12)	68.1 (11.1)	48.8 (11.6)	63.5 (11.5)
	Median	64.0	65.5	73.5	73.0	48.0	64.0
	Range	59–69	58–76	56–80	53–79	34–64	34–80
Gender, *n* (%)	Female	2 (25.0)	4 (50.0)	3 (37.5)	0	1 (12.5)	10 (25.6)
	Male	6 (75.0)	4 (50.0)	5 (62.5)	7 (100)	7 (87.5)	29 (74.4)
Ethnicity, *n* (%)	Hispanic or Latino	4 (50.0)	3 (37.5)	4 (50.0)	5 (71.4)	1 (12.5)	17 (43.6)
	Not Hispanic or Latino	4 (50.0)	5 (62.5)	4 (50.0)	2 (28.6)	7 (87.5)	22 (56.4)
Race, *n* (%)	White	7 (87.5)	6 (75.0)	7 (87.5)	7 (100)	0	27 (69.2)
	Black or African American	1 (12.5)	2 (25.0)	1 (12.5)	0	7 (87.5)	11 (28.2)
	Multiple^*b*^	0	0	0	0	1 (12.5)	1 (2.60)
BMI (kg/m^2^)	Mean (SD)	28.5 (2.53)	26.9 (3.00)	30.2 (4.05)	31.4 (2.24)	30.9 (5.12)	29.5 (3.79)
	Median	28.6	26.5	30.3	31.5	30.8	29.7
	Range	25.4–32.0	23.4–31.9	24.5–37.0	28.7–35.3	21.6–37.4	21.6–37.4
eGFR (mL/min/1.73 m^2^)	Mean (SD)	91.9 (11.3)	70.3 (9.97)	38.1 (8.80)	19.8 (4.47)	6.25 (1.58)	45.9 (33.2)
	Median	92.4	68.2	36.8	21.0	6.50	37.6
	Range	72.0–110	60.0–89.8	30.0–58.3	15.1–27.3	4.00–8.00	4.00–110
CL_CR_ (mL/min)	Mean (SD)	105 (13.9)	73.9 (12.8)	45.7 (8.79)	29.4 (9.68)	11.5 (2.88)	53.6 (35.0)
	Median	105	73.8	42.6	27.8	12.0	50.2
	Range	87.6–128	57.0–91.4	36.4–61.6	21.1–50.2	7.30–15.4	7.30–128

^*a*^ Cohort 1, normal renal function; Cohort 2, mild RI; Cohort 3, moderate RI; Cohort 4, severe RI; Cohort 5, ESRD requiring hemodialysis. RI, renal impairment; ESRD, end-stage renal disease requiring hemodialysis (HD); BMI, body mass index; CLcr, creatinine clearance; eGFR, estimated glomerular filtration rate; *N*, number of subjects; *n*, number of non-missing observations; SD, standard deviation.

^*b*^ Participant belonging to more than one race category.

### Plasma pharmacokinetics

**Fig 1:**
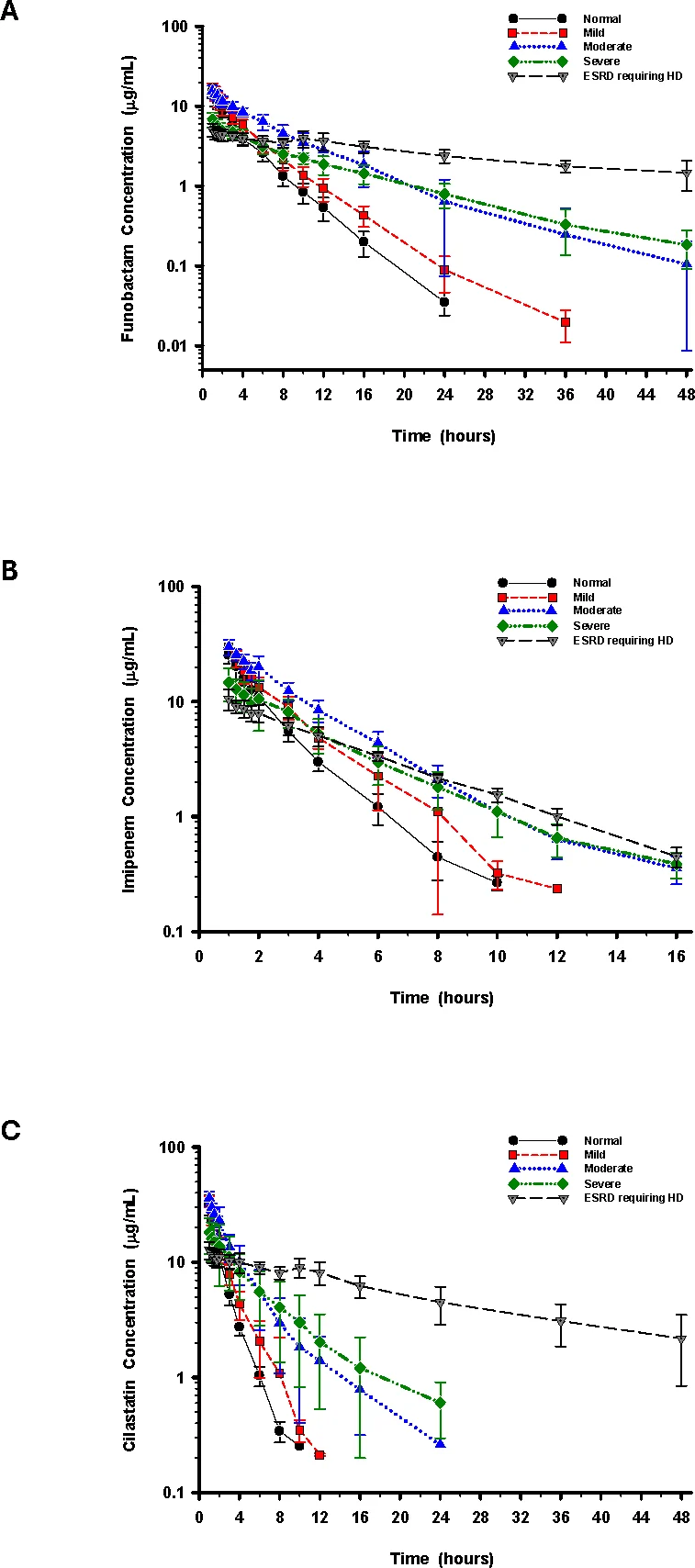
Arithmetic mean (±SD) plasma concentration versus time profiles of (**A**) funobactam, (**B**) imipenem, and (**C**) cilastatin following the 1-h intravenous infusion of 250 mg funobactam in combination with 500 mg imipenem/500 mg cilastatin (normal renal function and mild and moderate renal impairment) or 100 mg funobactam in combination with 200 mg imipenem/200 mg cilastatin (severe renal impairment and ESRD requiring dialysis). The y-axis is a semi-log scale.

Mean (SD) plasma concentration-time profiles following the 1-h intravenous infusion of 250 mg funobactam in combination with 500 mg imipenem/500 mg cilastatin (Cohorts 1–3) or 100 mg funobactam in combination with 200 mg imipenem/200 mg cilastatin (Cohorts 4 and 5) are displayed in Fig. 1. Plasma concentrations of funobactam, imipenem, and cilastatin were below the limit of quantitation in all participants prior to the start of infusion. Plasma concentrations of funobactam were measurable up to the 24-h sampling time point in all participants. Six participants in Cohort 3 and all participants in Cohorts 4 and 5 had their last quantifiable plasma concentrations of funobactam at the 48-h sampling time. Plasma concentrations of imipenem were measurable up to the 8-h sampling time in all participants. All participants in Cohort 5 and eight participants in Cohorts 3 and 4 (four participants in each cohort) had quantifiable plasma concentrations of imipenem up to the 16-h sampling time. All participants had measurable plasma concentrations of cilastatin up to the 8-h sampling time. All participants (*n* = 8) in Cohort 5 had quantifiable plasma concentrations of cilastatin up to the 48-h sampling time. One subject in Cohort 4 was retained in the data analyses despite having higher plasma concentrations of imipenem and cilastatin (and increased exposure parameters [maximum concentration {C_max_} and AUC values]) compared to other participants.

Mean (SD) pharmacokinetic parameters for funobactam, imipenem, and cilastatin in plasma are summarized per Cohort in Table 2. Variability of plasma pharmacokinetic parameters for funobactam was <25% in all cohorts. The largest intersubject variability (e.g., 30%–50% in geometric coefficient of variation [CV%]) of the pharmacokinetic parameter values was observed in Cohort 4 for imipenem and Cohorts 3, 4, and 5 for cilastatin.

**TABLE 2:** Pharmacokinetic parameters after a single 1-h intravenous infusion of 250 mg funobactam in combination with 500 mg imipenem/500 mg cilastatin (normal renal function and mild and moderate renal impairment) or 100 mg funobactam in combination with 200 mg imipenem/200 mg cilastatin (severe renal impairment and ESRD requiring hemodialysis)^*a*^

Analyte	Pharmacokinetic parameter (unit)	Normal renal function (*N* = 8)	Renal impairment			
			Mild (*N* = 8)	Moderate (*N* = 8)	Severe (*N* = 7)	ESRD (*N* = 8)
Funobactam	C_max_ (µg/mL)	15.3 (2.72)	16.7 (2.39)	15.8 (2.45)	6.89 (1.29)	5.18 (0.97)
	T_max_ (h)	1.00 (1.00, 1.02)	1.00 (1.00, 1.05)	1.00 (1.00, 1.50)	1.00 (1.00, 3.00)	1.00 (1.00, 10.0)
	AUC_0-last_ (µg*h/mL)	46.6 (7.68)	61.4 (10.9)	105 (25.0)	64.6 (13.6)	125 (16.8)
	AUC_0-∞_ (µg*h/mL)	46.7 (7.73)	61.6 (10.8)	106 (26.1)	67.6 (14.9)	180 (34.9)
	t_1/2_ (h)	2.89 (0.11)	3.62 (0.46)	6.05 (1.77)	10.2 (1.95)	25.7 (4.15)
	CL (L/h)	5.52 (1.20)	4.18 (0.75)	2.47 (0.57)	1.55 (0.39)	0.58 (0.11)
	V_Z_ (L)	22.9 (3.98)	21.8 (4.54)	20.5 (3.18)	22.2 (3.44)	21.1 (4.01)
Imipenem	C_max_ (µg/mL)	25.5 (3.87)	30.0 (4.61)	30.3 (4.20)	14.8 (5.58)	10.4 (2.06)
	T_max_ (h)	1.00 (1.00, 1.25)	1.00 (1.00, 1.05)	1.00 (1.00, 2.00)	1.00 (1.00, 1.50)	1.00 (1.00, 1.25)
	AUC_0-last_ (µg*h/mL)	47.2 (6.81)	63.4 (11.9)	87.5 (11.1)	53.6 (29.3)	49.2 (6.63)
	AUC_0-∞_ (µg*h/mL)	47.8 (6.85)	66.4 (17.2)	88.8 (11.5)	54.9 (30.2)	51.5 (6.94)
	t_1/2_ (h)	1.39 (0.16)	1.84 (0.87)	2.15 (0.47)	2.62 (0.37)	3.42 (0.19)
	CL (L/h)	10.7 (1.62)	7.89 (1.62)	5.72 (0.79)	4.31 (1.57)	3.95 (0.53)
	V_Z_ (L)	21.3 (3.30)	19.5 (4.10)	17.5 (3.80)	15.6 (4.40)	19.5 (2.90)
Cilastatin	C_max_ (µg/mL)	32.0 (6.25)	33.8 (3.79)	35.9 (5.39)	18.1 (6.18)	12.8 (2.06)
	T_max_ (h)	1.00 (1.00, 1.02)	1.00 (1.00, 1.05)	1.00 (1.00, 2.00)	1.00 (1.00, 1.50)	1.00 (1.00, 1.25)
	AUC_0-last_ (µg*h/mL)	51.2 (7.14)	63.7 (9.47)	108 (31.1)	91.6 (48.9)	254 (50.5)
	AUC_0-∞_ (µg*h/mL)	51.8 (7.05)	66.1 (12.4)	109 (31.7)	94.8 (50.3)	327 (111)
	t_1/2_ (h)	1.31 (0.13)	1.59 (0.44)	2.34 (0.83)	3.92 (0.84)	19.9 (6.98)
	CL (L/h)	9.79 (1.24)	7.77 (1.32)	4.94 (1.45)	2.57 (1.11)	0.67 (0.21)
	V_Z_ (L)	18.7 (3.62)	17.3 (2.44)	15.3 (2.90)	13.6 (4.00)	17.5 (2.35)

^*a*^ Cohort 1, normal renal function; Cohort 2, mild RI; Cohort 3, moderate RI; Cohort 4, severe RI; Cohort 5, ESRD requiring hemodialysis. RI, renal impairment; ESRD, end-stage renal disease requiring hemodialysis (HD); C_max_, maximum plasma concentration; T_max_, time to C_max_; area under the concentration-time curve (AUC) time 0 to the last quantifiable concentration (AUC_0-last_) and time 0 to infinity (AUC_0-∞_); t_1/2_, elimination half-life; CL, clearance; V_Z_, volume of distribution during terminal elimination phase. Arithmetic mean (SD) is presented for C_max_, AUC_0-last_, AUC_0-∞_, t_1/2_, CL and V_Z_. Median (minimum, maximum) is reported for T_max_.

### Effect of renal impairment on funobactam pharmacokinetic parameters

**Fig 2:**
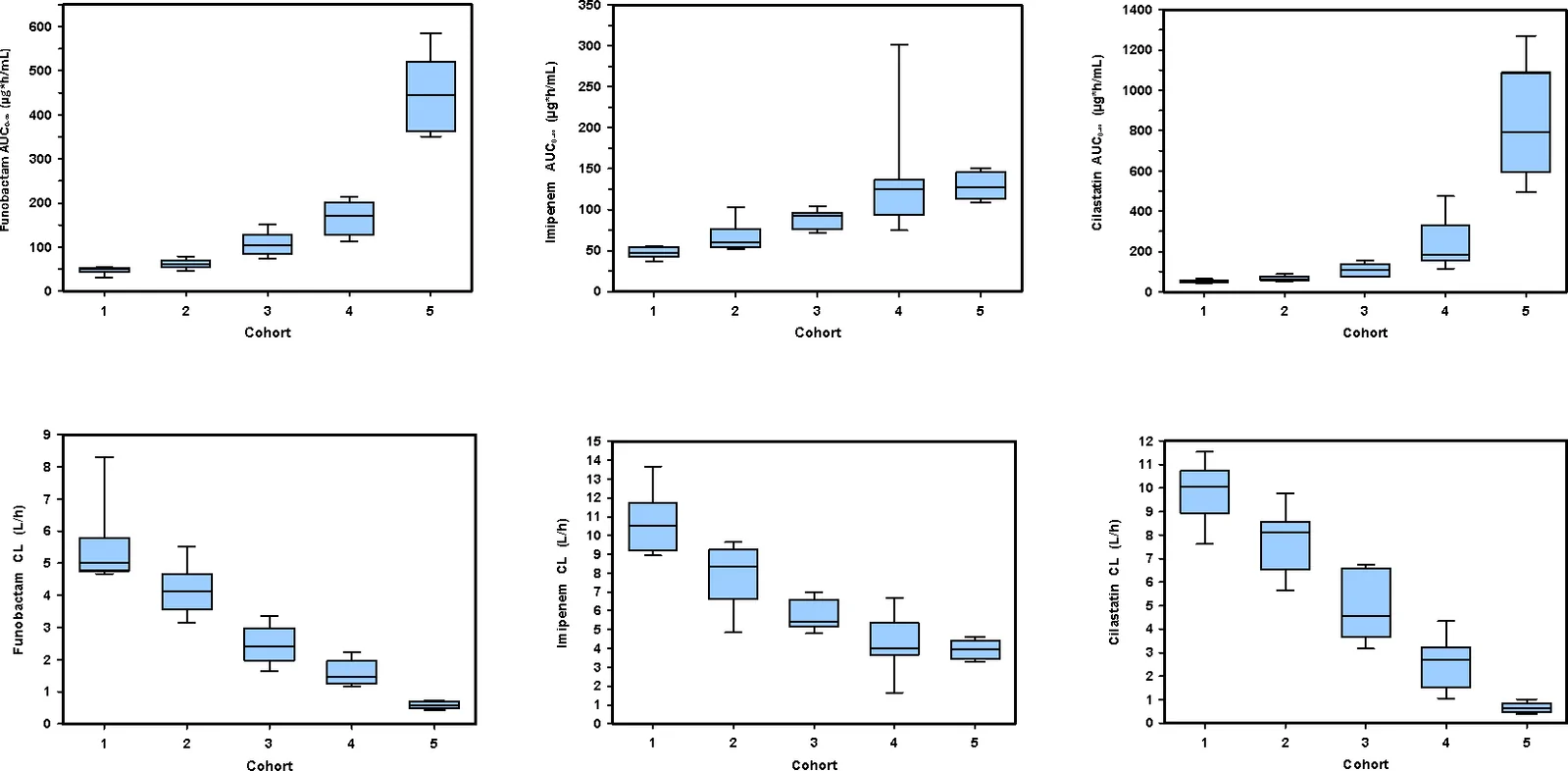
Box plots of AUC_0-∞_ (top row) and clearance (CL) (bottom row) of funobactam, imipenem, and cilastatin. The AUC_0-∞_ parameters for Cohorts 4 and 5 were scaled by 2.5 to match the same dose administered to Cohorts 1–3. In the box plot, the solid line is the median; the ends of the “box” are the 25th and 75th percentiles, and the whiskers show that the lowest data value is still within 1.5 IQR of the lower quartile. The highest value is still within 1.5 IQR of the upper quartile, where IQR is the interquartile range. Cohort 1, normal renal function; Cohort 2, mild renal impairment; Cohort 3, moderate renal impairment; Cohort 4, severe renal impairment; and Cohort 5, end-stage renal disease requiring hemodialysis.

Following single-dose administration of 100 mg funobactam in combination with 200 mg imipenem/200 mg cilastatin in Cohorts 4 and 5, the mean C_max_ values scaled by 2.5 (to account for the dose difference with Cohorts 1–3) were 17.2 and 12.9 µg/mL, respectively. After scaling, the mean C_max_ values across Cohorts 1–5 were comparable, except for Cohort 5, for which the C_max_ had a slightly decreasing trend. After scaling the mean AUC_0-∞_ values by 2.5 for Cohorts 4 and 5, the mean AUC_0-∞_ values of funobactam were 46.7, 61.6, 106, 169, and 450 µg*h/mL for Cohorts 1–5, respectively. Compared to participants with normal renal function (Cohort 1), least squares geometric mean ratios of AUC_0-∞_ for funobactam were 1.32-fold, 2.25-fold, 3.58-fold, and 9.58-fold higher in participants with mild RI (Cohort 2), moderate RI (Cohort 3), severe RI (Cohort 4), and participants with ESRD requiring HD (Cohort 5) (Table 3; Table S1). The estimated mean CL values of funobactam were 5.52, 4.18, 2.47, 1.55, and 0.58 L/h for Cohorts 1–5, respectively. Figure 2 illustrates the overall impact that renal impairment had on AUC_0-∞_ and CL of funobactam. Regression analysis of funobactam CL versus CL_CR_ and unadjusted eGFR demonstrated a strong correlation coefficient of 0.97 and 0.96, respectively (Table 4; Table S2). The estimated mean values of the volume of distribution during the terminal elimination phase (V_Z_) were comparable across all cohorts, whereas mean t_1/2_ values progressively increased from 2.89 h to 25.7 h for Cohorts 1 and 5, respectively (Table 2). Because of the prolonged t_1/2_ in Cohort 5, the values of AUC_0-∞_ for funobactam in participants with ESRD requiring HD should be considered a preliminary estimate and interpreted with caution since the extrapolated area beyond the 48-h sampling time was more than 20% in seven participants.

**TABLE 3:** Geometric mean ratios and 90% confidence intervals of AUC_0-∞_ for funobactam, imipenem, and cilastatin in participants with mild, moderate, and severe renal impairment, and ESRD requiring hemodialysis^*a*^

Analyte	Statistic	Normal renal function (*N* = 8)	Renal impairment			
			Mild (*N* = 8)	Moderate (*N* = 8)	Severe (*N* = 7)	ESRD (*N* = 8)
Funobactam	LS geo mean (SE)	46.1 (1.08)	60.7 (1.08)	103 (1.08)	165 (1.08)	442 (1.07)
	LS geo mean ratio (90% CI)^*b*^		1.32 (1.04, 1.66)	2.25 (1.78, 2.84)	3.58 (2.81, 4.56)	9.58 (7.59, 12.10)
Imipenem	LS geo mean (SE)	47.4 (1.09)	64.7 (1.09)	88.1 (1.09)	125 (1.10)	128 (1.09)
	LS geo mean ratio (90% CI)^*b*^		1.37 (1.04, 1.79)	1.86 (1.42, 2.43)	2.63 (1.99, 3.48)	2.69 (2.06, 3.52)
Cilastatin	LS geo mean (SE)	51.4 (1.11)	65.2 (1.11)	105 (1.11)	213 (1.12)	779 (1.11)
	LS geo mean ratio (90% CI)^*b*^		1.27 (0.90, 1.78)	2.04 (1.46, 2.87)	4.15 (2.92, 5.90)	15.15 (10.8, 21.3)

^*a*^ Cohort 1, normal renal function; Cohort 2, mild RI; Cohort 3, moderate RI; Cohort 4, severe RI; Cohort 5, ESRD requiring hemodialysis. RI, renal impairment; ESRD, end-stage renal disease requiring hemodialysis (HD); LS, least square; geo mean, geometric mean; SE, standard error; CI, confidence interval. The AUC_0-∞_ parameters for Cohorts 4 and 5 were scaled by 2.5 to match the same dose administered for Cohorts 1–3 before performing the ANOVA analysis.

^*b*^ The LS geo mean ratio (90% CI) for each cohort was computed with respect to Cohort 1.

**TABLE 4:** Summary of regression analysis of clearance of funobactam, imipenem, and cilastatin and renal function variable

Analyte	Independent variable	Coefficient of correlation	Slope	95% CI for slope	*P*-value
Funobactam	eGFR^*a*^	0.96	0.05	0.05, 0.06	<0.001
	CL_CR_^*b*^	0.97	0.05	0.05, 0.06	<0.001
Imipenem	eGFR^*a*^	0.93	0.07	0.06, 0.08	<0.001
	CL_CR_^*b*^	0.93	0.07	0.06, 0.08	<0.001
Cilastatin	eGFR^*a*^	0.96	0.09	0.08, 0.10	<0.001
	CL_CR_^*b*^	0.96	0.10	0.09, 0.11	<0.001

^*a*^ eGFR, estimated glomerular filtration rate calculated by the MDRD formula, transformed to unadjusted eGFR, and with units of mL/min.

^*b*^ CL_CR_, estimated creatinine clearance calculated by the Cockcroft-Gault equation and with units of mL/min.

### Effect of renal impairment on imipenem pharmacokinetic parameters

The mean C_max_ values of imipenem scaled by 2.5 for Cohorts 4 and 5 were 37.0 and 26.0 µg/mL, respectively. The mean C_max_ values across Cohorts 1–5 were comparable, with a slight increase in Cohort 4 participants. Mean AUC_0-∞_ values of imipenem (after dose scaling Cohorts 4 and 5) were 46.7, 61.6, 106, 169, and 450 µg*h/mL, for Cohorts 1–5, respectively. Least squares geometric mean ratios of AUC_0-∞_ of imipenem in Cohorts 2–5 were 1.37-fold, 1.86-fold, 2.63-fold, and 2.69-fold higher, respectively, compared to Cohort 1 (Table 3; Table S3). The estimated mean CL values (after dose scaling Cohorts 4 and 5) of imipenem for Cohorts 1–5 were 10.7, 7.89, 5.72, 4.31, and 3.95 L/h, respectively. Regression analysis of imipenem CL versus CL_CR_ and unadjusted eGFR demonstrated correlation coefficients of 0.93 (Table 4; Table S4). The overall impact of renal impairment on AUC_0-∞_ and CL of imipenem is illustrated in Fig. 2. The estimated mean values of V_Z_ of imipenem slightly decreased across Cohorts 1–4. Mean t_1/2_ values gradually increased from 1.39 to 3.42 h for Cohorts 1 and 5, respectively (Table 2).

### Effect of renal impairment on cilastatin pharmacokinetic parameters

The mean C_max_ values of cilastatin scaled by 2.5 for Cohorts 4 and 5 were 45.3 and 32.0 µg/mL, respectively. The mean C_max_ values across Cohorts 1, 2, 3, and 5 were comparable, with a 1.4-fold increase in Cohort 4 when compared to Cohort 1 participants. After scaling the AUC_0-∞_ values of Cohort 4 and 5 by 2.5, the mean AUC_0-∞_ values of cilastatin were 51.8, 66.1, 109, 237, and 818 µg*h/mL, for Cohorts 1–5, respectively, indicating 1.27-fold, 2.04-fold, 4.15-fold, and 15.15-fold increases in AUC_0-∞_ for participants with mild RI, moderate RI, severe RI, and participants with ESRD requiring HD, respectively, compared to the participants with normal renal function (Table 3; Table S5). The estimated mean CL values of cilastatin were 9.79, 7.77, 4.94, 2.57, and 0.67 L/h for Cohorts 1–5, respectively. The overall impact of renal impairment on AUC_0-∞_ and CL of cilastatin is illustrated in Fig. 2. The correlation coefficients between cilastatin CL versus CL_CR_ and unadjusted eGFR were 0.96 (Table 4; Table S6). The estimated mean of V_Z_ values for cilastatin across all cohorts were comparable, with a slight decrease in Cohort 4 participants. Mean t_1/2_ values of cilastatin progressively increased from 1.31 to 19.9 h for Cohorts 1 and 5, respectively (Table 2). Because of the prolonged t_1/2_ in Cohort 5, values of AUC_0-∞_ for cilastatin in participants with ESRD requiring HD should be interpreted with caution since the extrapolated area beyond the 48-h sampling time was more than 20% in three participants.

### Urinary pharmacokinetics

Thirty-one participants were included in the urine pharmacokinetic population. None of the eight participants with ESRD requiring HD were able to produce urine and were therefore excluded from the urine pharmacokinetic analysis. All participants (*N* = 31) with urine collection had pre-dose urine concentrations below the limit of quantitation. Urine pharmacokinetic parameters of funobactam, imipenem, and cilastatin for Cohorts 1–4 are summarized in Table 5.

**TABLE 5:** Pharmacokinetic parameters in urine after a single 1-h intravenous infusion of 250 mg funobactam in combination with 500 mg imipenem/500 mg cilastatin (normal renal function and mild and moderate renal impairment) or 100 mg funobactam in combination with 200 mg imipenem/200 mg cilastatin (severe renal impairment)^*a*^

Analyte	Pharmacokinetic parameter (unit)	Normal renal function (*N* = 8)	Renal impairment		
			Mild (*N* = 8)	Moderate (*N* = 8)	Severe (*N* = 7)
Funobactam	Ae (mg)	178 ± 43.1	197 ± 26.2	179 ± 39.8	59.1 ± 14.1
	Ae% (%)	71.4 ± 17.2	78.8 ± 10.5	71.5 ± 15.9	59.1 ± 14.1
	CL_R_ (L/h)	3.93 ± 1.12	3.34 ± 0.90	1.82 ± 0.68	0.94 ± 0.37
Imipenem	Ae (mg)	226 ± 100	254 ± 51.8	166 ± 71.0	55.9 ± 19.7
	Ae% (%)	45.2 ± 20.0	50.8 ± 10.4	33.2 ± 14.2	27.9 ± 9.83
	CL_R_ (L/h)	4.95 ± 2.40	4.03 ± 1.20	1.94 ± 0.98	1.16 ± 0.54
Cilastatin	Ae (mg)	352 ± 125	350 ± 67.7	355 ± 65.4	147 ± 70.5
	Ae% (%)	70.3 ± 25.0	70.0 ± 13.5	71.1 ± 13.1	73.5 ± 35.3
	CL_R_ (L/h)	6.98 ± 2.75	5.46 ± 1.39	3.62 ± 1.52	1.70 ± 0.60

^*a*^ Ae, cumulative amount of urinary excretion during post-dose urine sample collection intervals from 0 to 48 h; Ae%, fraction of drug excreted during post-dose urine sample collection intervals from 0 to 48 h, expressed as a percentage of the dose administered; CL_R_, renal clearance. Cohort 1, normal renal function; Cohort 2, mild renal impairment (RI); Cohort 3, moderate RI; Cohort 4, severe RI.

**Fig 3:**
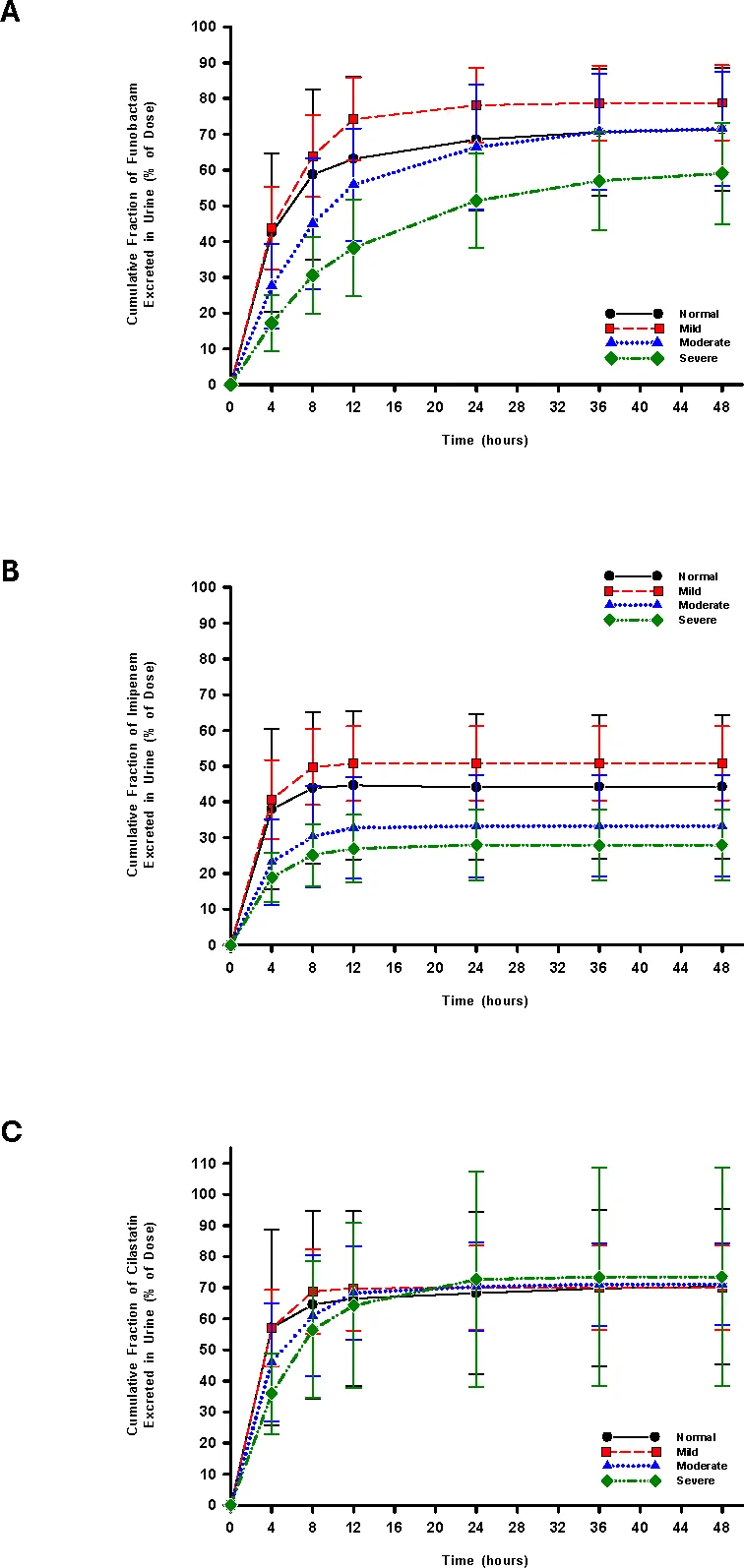
Arithmetic mean (±SD) cumulative fraction of urinary excretion during the 48-h collection (expressed as a percentage of the dose administered) of (**A**) funobactam, (**B**) imipenem, and (**C**) cilastatin for normal renal function and mild, moderate, and severe renal impairment.

The mean percentages of cumulative urine excretion (Ae%) of funobactam within 12 h were 63.1%, 74.2%, 55.9%, and 38.2% for Cohorts 1–4, respectively (Fig. 3), indicating the existence of pronounced renal excretion after 12 h. In comparison, the Ae% of funobactam were 71.4%, 78.8%, 71.5%, and 59.1% for Cohorts 1–4, respectively (Table 5). For imipenem, the Ae% for Cohorts 1–4 were 45.2%, 50.8%, 33.2%, and 27.9%, respectively. The mean Ae% values for cilastatin were similar across the four Cohorts (range: 70.0%–73.5%).

Renal clearance (CL_R_) of funobactam, imipenem, and cilastatin decreased as renal impairment increased. Mean CL_R_ values ranged from 3.93 to 0.94 L/h for funobactam, 4.95 to 1.16 L/h for imipenem, and 6.98 to 1.70 L/h for cilastatin (Table 5).

### Safety and tolerability

Co-administration of funobactam with imipenem and cilastatin was well tolerated. Three participants (7.7%) experienced a total of six treatment-emergent adverse events over the course of the study (Table 6). Five adverse events (AEs) were mild, and one AE was moderate in severity. Two participants with mild RI reported three adverse events (nausea [*n* = 1] and headache [*n* = 2]) that were considered probably related to the study treatment. One participant with ESRD experienced three AEs (diarrhea, hypotension, and COVID-19) that were considered unlikely related to the administration of the study treatment. There were no deaths, SAEs, or discontinuations due to AEs reported during the study.

**TABLE 6:** Summary of adverse events by system organ class and preferred term^*a*^

Adverse event	Normal renal function (*N* = 8) *N* (%)	Renal impairment				Overall (*N* = 39) *N* (%)
		Mild (*N* = 8) *n* (%)	Moderate (*N* = 8) *n* (%)	Severe (*N* = 7) *n* (%)	ESRD (*N* = 8) *n* (%)	
Total no. of adverse events	0	3	0	0	3	6
No. of subjects with at least one adverse event	0	2 (25.0)	0	0	1 (12.5)	3 (7.7)
Gastrointestinal disorders	0	1 (12.5)	0	0	1 (12.5)	2 (5.1)
Diarrhea	0	0	0	0	1 (12.5)	1 (2.6)
Nausea	0	1 (12.5)	0	0	0	1 (2.6)
Nervous system disorders	0	2 (25.0)	0	0	0	2 (5.1)
Headache	0	2 (25.0)	0	0	0	2 (5.1)
Infections and infestations	0	0	0	0	1 (12.5)	1 (2.6)
COVID-19	0	0	0	0	1 (12.5)	1 (2.6)
Vascular disorders	0	0	0	0	1 (12.5)	1 (2.6)
Hypotension	0	0	0	0	1 (12.5)	1 (2.6)

^*a*^ Cohort 1, normal renal function; Cohort 2, mild RI; Cohort 3, moderate RI; Cohort 4, severe RI; Cohort 5, ESRD requiring hemodialysis. RI, renal impairment; ESRD, end-stage renal disease requiring hemodialysis (HD).

There were no clinically significant abnormalities in the clinical laboratory tests (hematology, biochemistry, coagulation, urinalysis, and urine microscopy) during the study. There were no abnormal or clinically significant physical examination findings reported during the study. The mean changes from baseline in the vital signs and electrocardiogram (ECG) findings were not notably different at various time points for all enrolled participants. A few participants had abnormal vital sign measurements and ECG findings at various time points; however, there were no clinically significant findings as judged by the investigator.

## DISCUSSION

This study evaluated the plasma and urine pharmacokinetic profiles of funobactam, imipenem, and cilastatin in participants with normal and impaired renal function. Thirty-nine (*N* = 39) participants were enrolled in the study, and all participants received a single dose of funobactam in combination with imipenem and cilastatin, given as a 60-min intravenous infusion. In this study, healthy control participants with normal renal function (Cohort 1) were enrolled and matched to a rolling pooled mean for BMI, sex (ratio of males versus females), and mean age observed in enrolled participants in Cohorts 3 and 4.

Cohort 1 included six male and two female participants with a mean (±SD) age of 64 (±3.8) years. The estimated mean C_max_, AUC_0-∞_, CL, V_Z_, and t_1/2_ for funobactam in Cohort 1 were 15.3 µg/mL, 46.7 µg*h/mL, 5.52 L/h, 22.9 L, and 2.89 h, respectively, which is comparable to the values obtained from the previous study in healthy young male participants following 250 mg single-dose administration of funobactam (13.3 µg/mL, 44.7 µg*h/mL, 5.71 L/h, 24.3 L, and 3.0 h, respectively) (4). Similarly, the estimated mean C_max_, AUC_0-∞_, CL, V_Z_, and t_1/2_ values for imipenem/cilastatin were 25.5/32.0 µg/mL, 47.8/51.8 µg*h/mL, 10.7/9.79 L/h, 21.3/18.7 L, and 1.39/1.31 h, respectively, comparable to those obtained from the previous studies in healthy male and female participants (age: 18–40 years) and healthy adult elderly male and female participants (age: 60–75 years) following single 500 mg imipenem/500 mg cilastatin dose administration (11, 12). Our results for pharmacokinetics of imipenem and cilastatin are consistent with those of previous investigations in participants with renal impairment (6, 12–14).

Although there was a progressive increase in mean AUC_0-∞_ when the degree of renal insufficiency increased from participants with mild renal impairment to participants with ESRD requiring HD, the mean C_max_ overall was similar among the five study cohorts. C_max_ was primarily impacted by the infusion rate, and the study drugs were delivered at the same rate after scaling Cohorts 4 and 5 to the same dose administered for Cohorts 1–3. In addition, apparent volumes of distribution were similar among the different cohorts, consistent with data on other β-lactam β-lactamase inhibitor combinations when the distribution is primarily to the human circulation system and extracellular fluid and not impacted by renal insufficiency (12, 13, 15–17).

Funobactam and cilastatin demonstrated a slightly greater extent of renal excretion than imipenem. Cohorts 1–3 had approximately 70% renal excretion for funobactam and cilastatin compared to approximately 50% renal excretion for imipenem. Our renal excretion results for imipenem are consistent with recent investigations in participants with normal renal function, however, lower than the previously reported value of 70% in earlier studies (5, 6, 11, 12). For Cohort 4, the excretion of funobactam and cilastatin was approximately twice that of imipenem. No confounding factors (e.g., incomplete urine collection) were observed to explain the lower cumulative urinary excretion (Ae%) of funobactam and imipenem for Cohort 4. The progressive decreases in cumulative urinary excretion of imipenem are consistent with those observed by Bhagunde et al. (12).

The analysis also demonstrated a similar high correlation (r ≥ 0.93) of CL of funobactam, imipenem, and cilastatin to individual eGFR or CL_CR_, indicating that either could be used for dose adjustment in participants with renal impairment. Because of the overall strong correlation between the CL of funobactam, imipenem, cilastatin, and renal function, the CL rates of funobactam, imipenem, and cilastatin progressively decreased with the increase in the degree of renal insufficiency in participants with mild, moderate, and severe renal impairment and in participants with ESRD requiring HD and resulted in a correspondingly progressive increase in the AUC_0-∞_. Dose adjustment will likely be needed in participants with mild, moderate, and severe renal impairment and in participants with ESRD requiring HD.

All 39 participants enrolled in this study were included in the evaluation of safety and tolerability. Following a single intravenous infusion, a total of six AEs (five mild and one moderate in severity) were observed in three participants (7.7%). No clinically significant findings or increasing or decreasing trends from baseline were observed for any of the safety parameters.

This study had several limitations. First, participants with ESRD requiring HD did not undergo hemodialysis in this study. Studies in participants undergoing hemodialysis will need to be conducted to determine the amount of funobactam that is dialyzable and to establish the dose and timing recommendations for the co-administration of funobactam and imipenem/cilastatin. Second, the study participants were not specifically matched according to race or ethnicity, resulting in seven of eight participants with ESRD requiring HD (Cohort 5) being Black, whereas the majority of participants in Cohorts 1–4 were White. Little information is available on whether race or ethnicity can significantly change the pharmacokinetics of funobactam, imipenem, or cilastatin. The likelihood that race or ethnicity influenced our pharmacokinetic results is minimal since demographic characteristics (age, sex, and BMI) were similar among all cohorts and marked differences in renal elimination occurred between ESRD participants and the other cohorts. Finally, the study’s evaluation of pharmacokinetics and safety was in participants without infections and/or unstable ESRD requiring HD, where other factors may influence the disposition of these agents.

In summary, a single 1-h intravenous infusion of funobactam combined with imipenem and cilastatin was safe and well tolerated in participants with normal and impaired renal function. The pharmacokinetics results indicated that plasma AUC_0-∞_ of funobactam, imipenem, and cilastatin increased with increasing renal impairment and was associated with decreased CL, CL_R_, and elimination t_1/2_. Renal impairment had minimal impact on C_max_, T_max_, and V_z_. These observations come as no surprise since a high percentage of drug elimination of funobactam (71.4%), imipenem (45.2%), and cilastatin (70.3%) was renal excretion in the participants with normal renal function. These results suggest adjustments in dose and/or dosing interval will be required in patients with various degrees of renal impairment.

## MATERIALS AND METHODS

The study was conducted at the Division of Clinical Pharmacology at the University of Miami, FL, and Orlando Clinical Research Center, Orlando, FL, in accordance with U.S. Food and Drug Administration regulations, ICH E6 (R2) Good Clinical Practice (GCP), and applicable local, state, and federal laws.

### Study design

**Fig 4:**
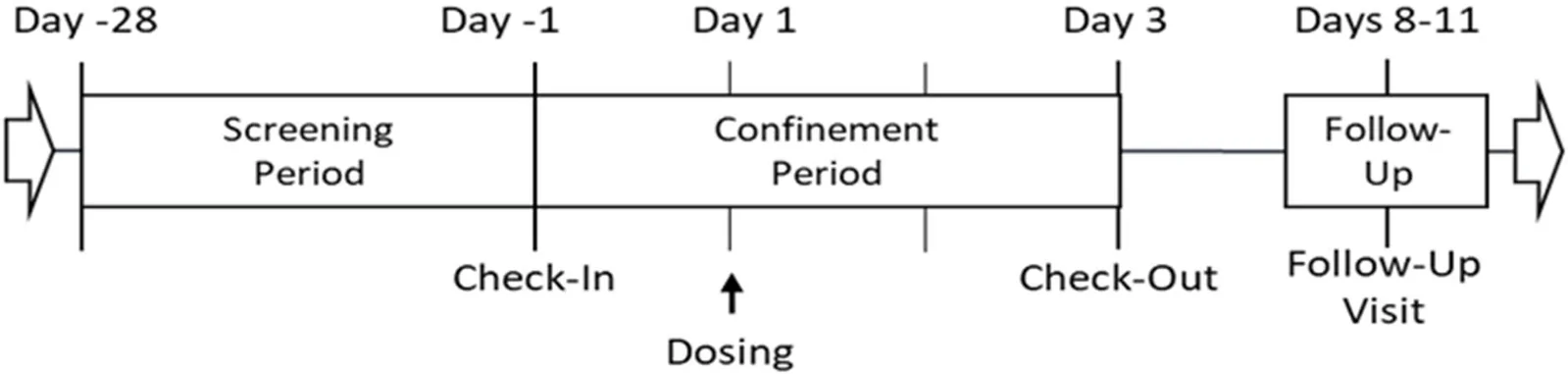
Study schematic.

This was a Phase 1, two-center, open-label study to assess the pharmacokinetics, safety, and tolerability of funobactam, imipenem, and cilastatin administered by intravenous infusion to adults with normal renal function and various degrees of renal impairment, including ESRD requiring HD (Fig. 4). Participants with ESRD requiring HD had pharmacokinetic study procedures conducted on a non-dialysis day. The design of this study followed recommendations for conducting a pharmacokinetic study of a new drug in subjects with renal impairment (15–17).

Participants were assigned to a renal function category based on estimated glomerular filtration rate (eGFR) using the modification of diet in renal disease (MDRD) equation (9) (eGFR = 175 × standardized Scr^-1.154^ × age^-0.203^ × 1.212 [if African American] × 0.742 [if female], where eGFR is in mL/min/1.73 m^2^, Scr is measured serum creatinine in mg/dL, and age is in years): Cohort 1, normal renal function (eGFR ≥90 mL/min/1.73 m^2^); Cohort 2, mild renal impairment (eGFR 60 to <90 mL/min/1.73 m^2^); Cohort 3, moderate renal impairment (eGFR 30 to <60 mL/min/1.73 m^2^); and Cohort 4, severe renal impairment (eGFR 15 to <30 mL/min/1.73 m^2^). Participants with stable ESRD requiring HD were assigned to Cohort 5 and received HD at least three times a week for at least 3 months prior to screening. Estimated creatinine clearance using the Cockcroft-Gault equation was also calculated for participants in Cohorts 1–4 at screening and used for data analysis and interpretation (10).

The study allowed simultaneous enrollment into Cohorts 2–5 first. After enrollment of at least 50% of participants in Cohorts 3 and 4 (8 of 16; four per cohort), matched controls were enrolled in Cohort 1. Healthy control participants in Cohort 1 were matched to a rolling pooled mean for BMI (± 20%), sex (similar ratio, 1:1 ± 1 subject), and mean age (± 10 years) observed in enrolled participants in Cohorts 3 and 4.

### Selection of participants

Eligible participants were adult men and women aged ≥18 years with a BMI between ≥18.5 and ≤39.9 kg/m^2^ and total body weight between 50.0 and 130.0 kg inclusive. Women were allowed to participate if they were postmenopausal or agreed to use birth control. To be included, the participants were required to be medically healthy without clinically significant abnormalities (Cohort 1 only) or medically stable without clinically significant acute or chronic illness (Cohorts 2–5) that may impact the assessment of pharmacokinetics and safety as assessed by the investigator based on screening medical history, physical examination, vital signs, 12-lead electrocardiogram (ECG), hematology, biochemistry, coagulation, and urinalysis. Women must have been non-pregnant or non-lactating and be either postmenopausal or agreed to use an acceptable form of contraception (as defined in the protocol) throughout the study and for 30 days after study completion. Male participants engaging in sexual activity were required to use two highly effective methods of birth control (as defined in the protocol) from the time of the screening visit until 90 days following the administration of funobactam, imipenem, and cilastatin. Male participants were not allowed to donate sperm during this time. Participants had to be willing to refrain from consumption of alcohol, caffeine, xanthine-containing beverages, or food or product containing any of these or product containing any of these from 48 h prior to study drug administration until time of discharge from the clinical study unit.

Participants were excluded if they had any clinically significant medical history or abnormal findings upon physical examination or clinical laboratory tests; ECG with a QTcF interval duration equal to or greater than 500 ms; a history of chronic liver disease, cirrhosis, or biliary disease; a history of or presence of CNS disorders, seizures, or other CNS adverse reactions; positive testing for severe acute respiratory syndrome coronavirus 2 (SARS-CoV-2); close contact with anyone who had tested positive for SARS-CoV-2 infection within 14 days before the screening visit; the presence of SARS-CoV-2 symptoms within 14 days prior to screening, at the screening visit, or check-in (Day −1); and a recent history (within 6 months) of known or suspected *Clostridium difficile* infection.

Exclusion criteria also included the following: (i) a positive known history of clinically significant hypersensitivity reaction or anaphylaxis to any medication, (ii) a history of clinically significant hypersensitivity to the study drugs or any β-lactam agents or to any of the excipients, (iii) a history of food intolerance, (iv) positive testing for human immunodeficiency virus antibody, hepatitis B surface antigen, or hepatitis C virus antibody, (v) a recent history of substance or alcohol abuse within the previous year, (vi) positive drug screen and alcohol testing at screening or check-in visit (Day −1), (vii) habitual use of tobacco or nicotine products or smoking within 3 months prior to screening, (viii) participants with normal renal function (Cohort 1), the use of any over-the-counter medications within 7 days prior to study drug administration or use of prescription medications including nonsteroidal anti-inflammatory drugs, health supplements, and herbal remedies taken within 14 days prior to study drug administration, (ix) participants with renal impairment (Cohorts 2–5), the use of prohibited concomitant medication with the exception of those essential for the management of renal impairment and other concomitant stable medical conditions as per the discretion of the investigator, (x) use of probenecid or valproic acid within 30 days prior to study drug administration, (xi) receipt of an investigational drug within 30 days or five half-lives prior to the first administration of study drug, whichever is longer, and (xii) donation of blood or plasma within 30 days prior to dosing, loss of whole blood of more than 500 mL within 30 days prior to dosing, or receipt of a blood transfusion within 1 year of study enrollment.

### Safety assessments

All safety and tolerability parameters were monitored throughout the duration of the study. Study assessments included complete physical examinations, vital sign measurements (blood pressure, heart rate, respiratory rate, and oral temperature), 12-lead ECGs, clinical laboratory tests (e.g., hematology, biochemistry, coagulation, and urinalysis), and monitoring of AEs.

### Drug administration

Participants in Cohorts 1–3 were administered a single dose of 250 mg funobactam in combination with 500 mg imipenem and 500 mg cilastatin by intravenous infusion administered over 60 min on Day 1. Participants in Cohorts 4 and 5 were administered a single dose of 100 mg funobactam in combination with 200 mg imipenem and 200 mg cilastatin by intravenous infusion administered over 60 min on Day 1. Participants with ESRD receiving HD were administered the study drug on a non-dialysis day. Funobactam and imipenem/cilastatin infusions were initiated at the same time.

The dose selection of imipenem/cilastatin/funobactam was based on established renal dose adjustment guidelines for imipenem/cilastatin/relebactam because both combination products share the same β-lactam partner (imipenem/cilastatin) and both β-lactamase inhibitors (funobactam and relebactam) are from the same class of diazabicyclooctane β-lactamase inhibitor (4, 11, 12, 18). After Phase I SAD/MAD studies, funobactam displayed similar pharmacokinetic properties to relebactam (linear pharmacokinetics and elimination by the kidney), although funobactam showed a slightly longer elimination half-life (4). These similarities allowed selected doses of imipenem/cilastatin/funobactam to be the same as imipenem/cilastatin/relebactam for Cohorts 1, 4, and 5. Modest dose adjustments are recommended for imipenem/cilastatin/relebactam for Cohorts 2 and 3 (imipenem 400 mg/cilastatin 400 mg/relebactam 200 mg for Cohort 2, and imipenem 300 mg/cilastatin 300 mg/relebactam 150 mg for Cohort 3) (18). This study decided not to adjust the doses of imipenem/cilastatin/funobactam for Cohorts 2 and 3 because dose reduction of imipenem/cilastatin/relebactam was relatively small, the study involves a single dose, all three components have short elimination half-lives, and there is low potential for safety concerns with slightly higher exposure. In addition, the renal impairment study is exploratory in nature and focused primarily on characterizing pharmacokinetics of funobactam, imipenem, and cilastatin.

### Blood collection

Serial blood samples for determination of funobactam, imipenem, and cilastatin plasma concentrations were collected at the following time points: 0 h (pre-dose) and 1, 1.25, 1.5, 1.75, 2, 3, 4, 6, 8, 10, 12, 16, 24, 36, and 48 h after the start of infusion. All blood samples were collected on non-dialysis days in participants with ESRD requiring HD.

### Urine collection

For participants in Cohorts 1–4, total voided urine collection was over a 4-h interval before dosing (–4 to 0 h) and at 0–4, 4–8, 8–12, 12–24, 24–36, and 36–48 h after dosing. Urine collection for urinalysis (cohorts 4 and 5) or urine pharmacokinetic analysis (cohort 4) was waived in participants who could not provide a urine sample due to the lack of urine production.

### Determination of funobactam, imipenem, and cilastatin concentrations

Plasma and urine concentrations of funobactam, imipenem, and cilastatin were determined by validated liquid chromatography with tandem mass spectrometry (LC-MS/MS) assays at Pharmaron ABS (Germantown, MD). A total of 623 plasma and 217 urine samples were assayed for funobactam concentrations (method number 209M001) and imipenem and cilastatin concentrations (method number 180M005).

### Determination of funobactam concentrations in plasma

In brief, the method for funobactam used protein precipitation (methanol was used as the precipitation solvent) to isolate funobactam and the internal standards (funobactam-^13^C_3_-^15^N [Bellen, lot no. 60401-My-026]). Pre-treated human K_2_EDTA plasma was used as a blank matrix. Human K_2_EDTA plasma was pretreated by mixing one part of stabilizer (ethylene glycol:water:1 M pH 6.0 2-(N-Morpholino)-Ethanesulfonic [MES], 25:25:50 [vol/vol/vol]) with one part of human K_2_EDTA plasma (50:50, vol/vol). Pre-treated human K_2_EDTA plasma was spiked with the appropriate concentration of working standard solution to give the calibration standards and quality control (QC) samples. After shaking and centrifuging, 50 μL of the supernatant was transferred into the 96-well plate containing 150 μL of acetonitrile, and the plate was then vortexed for 5 min. A total of 5 μL injection volume was used for LC-MS/MS analysis.

The standard curves were linear for funobactam (R^2^ ≥ 0.9954) over the concentration ranges of 5–5,000 ng/mL. The respective intra-run precision and accuracy for funobactam QC samples were 2.14%–3.21% and 1.60%–9.00% at 5 ng/mL, 1.41%–4.99% and 2.67%–12.00% at 15 ng/mL, 2.63%–3.12% and 3.25%–10.25% at 400 ng/mL, and 2.33%–4.64% and –2.00% to 0.50% at 4,000 ng/mL. The respective inter-run precision and accuracy for funobactam QC samples were 4.09% and 6.20% at 5 ng/mL, 5.62% and 6.00% at 15 ng/mL, 3.96% and 6.00% at 400 ng/mL, and 3.48% and −0.75% at 4,000 ng/mL. A total of 63 samples were selected for incurred sample assay reproducibility (ISR) testing, and 96.8% of them met the acceptance criteria, with at least two-thirds of the repeat results and original results falling within 20% of the mean of the two values. The lower limit of quantification (LLOQ) was 5 ng/mL for plasma concentrations of funobactam. Reported concentrations were multiplied by a factor of 2 to account for the 1:1 dilution of plasma samples with stabilizer.

### Determination of imipenem and cilastatin concentrations in plasma

In brief, the method used protein precipitation (acetonitrile:methanol [50:50, vol/vol] was used as the precipitation solvent) to isolate imipenem, cilastatin, and the internal standards (Oseltamivir-d_3_ [TRC, lot no. 14-SBK-47-3]). Pre-treated human K_2_EDTA plasma was used as a blank matrix. Human K_2_EDTA plasma was pretreated by mixing one part of stabilizer (ethylene glycol:water:1 M pH 6.0 MES, 25:25:50 [vol/vol/vol]) with one part of human K_2_EDTA plasma (50:50, vol/vol). Pre-treated human K_2_EDTA plasma was spiked with the appropriate concentration of working standard solution to give the calibration standards and QC samples. After shaking and centrifuging, 200 μL of the supernatant was transferred into the 96-well plate. A total of 5 μL injection volume was used for LC-MS/MS analysis.

The standard curves were linear for imipenem (R^2^ ≥0.9979) and cilastatin (R^2^ ≥0.9958) over the concentration ranges of 100–50,000 ng/mL of both imipenem and cilastatin. The respective intra-run precision and accuracy for imipenem QC samples were 2.39%–5.24% and −9.70% to −3.00% at 100 ng/mL, 2.40%–4.05% and −5.33% to −5.00% at 300 ng/mL, 2.34%–4.73% and −11.00% to −3.25% at 4,000 ng/mL, and 2.73%–5.43% and −9.75% to −5.00% at 40,000 ng/mL. The respective inter-run precision and accuracy for imipenem QC samples were 5.13% and −5.70% at 100 ng/mL, 3.11% and −5.33% at 300 ng/mL, 4.85% and −7.25% at 4,000 ng/mL, and 4.35% and −7.00% at 40,000 ng/mL. The respective intra-run precision and accuracy for cilastatin QC samples were 2.64%–4.41% and −5.10% to 5.00% at 100 ng/mL, 3.70%–4.52% and −5.33% to 2.67% at 300 ng/mL, 2.56%–8.13%, and −3.25% to −2.25% at 4,000 ng/mL, and 1.76%–5.56% and −2.00% to −0.75% at 40,000 ng/mL. The respective inter-run precision and accuracy for cilastatin QC samples were 5.85% and 2.00% at 100 ng/mL, 5.19% and −1.67% at 300 ng/mL, 5.27% and −2.75% at 4,000 ng/mL, and 3.76% and −1.50% at 40,000 ng/mL. A total of 62 samples were selected for ISR testing, and 88.7% of imipenem and 90.3% of cilastatin met the acceptance criteria, respectively, with at least two-thirds of the repeat results and original results falling within 20% of the mean of the two values. The LLOQ was 100 ng/mL for plasma concentrations of both drugs. Reported concentrations were multiplied by a factor of 2 to account for the 1:1 dilution of plasma samples with stabilizer.

### Determination of funobactam concentrations in urine

In brief, the method used protein precipitation (methanol was used as the precipitation solvent) to isolate funobactam and the internal standards (funobactam-^13^C_3_-^15^N [Bellen, lot no. 60401-My-026]). Human urine was spiked with the appropriate concentration of working standard solution to give the calibration standards and QC samples. After shaking and centrifuging, 25 μL of the supernatant was transferred into the 96-well plate containing 175 μL of acetonitrile, and the plate was then vortexed for 5 min. A total of 5 μL injection volume was used for LC-MS/MS analysis.

The standard curves were linear for funobactam (R^2^ ≥0.9972) over the concentration ranges of 100–100,000 ng/mL. The respective intra-run precision and accuracy for funobactam QC samples were 3.64%–6.37% and 2.00%–11.00% at 100 ng/mL, 2.26%–7.24% and –0.33% to 11.67% at 3,000 ng/mL, 1.70%–3.64% and –2.75% to 8.00% at 8,000 ng/mL, and 2.46%–7.89% and 2.50%–10.25% at 80,000 ng/mL. The respective inter-run precision and accuracy for funobactam QC samples were 6.68% and 5.00% at 100 ng/mL, 6.89% and 4.00% at 300 ng/mL, 5.62% and 1.13% at 8,000 ng/mL, and 5.80% and 5.25% at 80,000 ng/mL. A total of 30 samples were selected for ISR testing, and 83.3% of them met acceptance criteria, with at least two-thirds of the repeat results and original results falling within 20% of the mean of the two values. The LLOQ was 100 ng/mL for urine concentrations of funobactam.

### Determination of imipenem and cilastatin concentrations in urine

In brief, the method used protein precipitation (acetonitrile:methanol [50:50, vol/vol] was used as the precipitation solvent) to isolate imipenem, cilastatin, and the internal standards (Oseltamivir-d_3_ [TRC, lot no. 14-SBK-47-3]). Human urine was spiked with the appropriate concentration of working standard solution to give the calibration standards and QC samples. After shaking and centrifuging, 200 μL of the supernatant was transferred into the 96-well plate. A total of 5 μL injection volume was used for LC-MS/MS analysis.

The standard curves were linear for imipenem (R^2^ ≥ 0.9902) and cilastatin (R^2^ ≥ 0.9926) over the concentration ranges of 500–50,000 ng/mL of both imipenem and cilastatin. The respective intra-run precision and accuracy for imipenem QC samples were 3.65%–5.65% and –9.00% to 4.00% at 500 ng/mL, 2.09%–2.63% and −4.67% to −4.00% at 1,500 ng/mL, 2.86%–5.38% and –1.25% to 2.75% at 4,000 ng/mL, and 2.68%–3.11% and –1.25% to 5.75% at 40,000 ng/mL. The respective inter-run precision and accuracy for imipenem QC samples were 7.30% and −3.60% at 500 ng/mL, 2.26% and −4.00% at 1,500 ng/mL, 4.26% and 0.25% at 4,000 ng/mL, and 4.14% and 1.50% at 40,000 ng/mL. The respective intra-run precision and accuracy for cilastatin QC samples were 4.10%–4.94% and –11.80% to 4.40% at 500 ng/mL, 2.37%–4.86% and –8.00% to 11.33% at 1,500 ng/mL, 2.28%–3.53% and –3.75% to 6.50% at 4,000 ng/mL, and 1.91%–2.86% and –1.00% to 1.25% at 40,000 ng/mL. The respective inter-run precision and accuracy for cilastatin QC samples were 8.11% and −3.60% at 500 ng/mL, 9.60% and −0.67% at 1,500 ng/mL, 5.46% and −0.25% at 4,000 ng/mL, and 2.42% and 0.25% at 40,000 ng/mL. A total of 34 samples were selected for ISR testing, and 88.2% of imipenem and 94.1% of cilastatin met the acceptance criteria, respectively, with at least two-thirds of the repeat results and original results falling within 20% of the mean of the two values. The LLOQ was 500 ng/mL for urine concentrations of both drugs.

### Pharmacokinetic analyses

The maximum plasma concentration (C_max_) and time to C_max_ (T_max_) were read from the observed plasma concentration-time profile after the start of infusions of funobactam, imipenem, and cilastatin. Non-compartmental methods were used to generate pharmacokinetic parameters in plasma and urine using Phoenix WinNonlin version 8.3 (Certara USA, Inc., Princeton, New Jersey). The area under the concentration-time curve (AUC) values were computed with the linear-log trapezoidal method and included time 0–48 h (AUC_0-48_), time 0 to the last quantifiable concentration (AUC_0-last_), and time 0 to infinity (AUC_0-∞_). Plasma pharmacokinetic parameters included CL, volume of distribution at steady state (V_SS_), and volume of distribution during the terminal elimination phase (V_Z_). The elimination rate constant (λ_z_) was determined by linear regression, and the elimination half-life (t_1/2_) was calculated with the equation t_1/2_ = 0.693/λ_z_.

The pharmacokinetic data in urine included the cumulative amount of urinary excretion during the 48-h collection (Ae), Ae expressed as a percentage of the dose administered (Ae%; calculated as Ae/Dose × 100%), and renal clearance (CL_R_; calculated as Ae/AUC_0-∞_).

### Statistical analysis

This was primarily a descriptive study evaluating funobactam, imipenem, and cilastatin plasma and urine concentrations and pharmacokinetic parameters. Pharmacokinetic parameters and concentrations for individual participants and the three analytes were summarized by cohort using descriptive statistics (sample size [n], arithmetic means, median, minimum, maximum, standard deviation, geometric mean, and geometric CV%). A sample size of eight participants per cohort was considered sufficient to provide adequate data for inclusion into a pharmacokinetic analysis (17). Eight participants were recruited in each cohort to ensure at least six evaluable participants per cohort.

Linear regression was used to model changes in pharmacokinetic parameters versus continuous independent variables representative of renal function (eGFR [in units of mL/min after correction by individual body surface area] and CL_CR_). Assessed pharmacokinetic parameters included C_max_, AUC_0-last_, AUC_0-∞_, and CL.

Statistical analysis was carried out using an analysis of variance (ANOVA) model on the log-transformed values of the pharmacokinetic parameters of C_max_, AUC_0-∞_, CL, V_ss_, V_Z_, and t_1/2_ as the response variables and with the fixed-effect term of cohort as a categorical variable. The estimated least squares (LS) mean difference and associated 90% CI were calculated for each RI group (Cohorts 2–5 versus participants with normal renal function [Cohort 1]) and were back-transformed to provide LS geometric mean ratios and 90% CIs for each comparison. Multiple comparisons were assessed using the Dunnett test.

## SUPPLEMENTAL MATERIAL

Supplemental tables aac.00808-26-s0001.docx Tables S1 to S6.

ASM does not own the copyrights to Supplemental Material that may be linked to, or accessed through, an article. The authors have granted ASM a non-exclusive, world-wide license to publish the Supplemental Material files. Please contact the corresponding author directly for reuse.
