# Supplementary material for: Impact of renal impairment on pharmacokinetics, safety, and tolerability of funobactam, combined with imipenem and cilastatin
Source: Antimicrob Agents Chemother. 2026 Aug 27;70(10):e00808-26. doi: 10.1128/aac.00808-26 (PMC13689130; doi:10.1128/aac.00808-26)
Supplement: Supplementary file 1 [file aac.00808-26-s0001.docx]

**Supplemental Tables**

**Impact of Renal Impairment on Pharmacokinetics, Safety and Tolerability**

**of Funobactam, Combined with Imipenem and Cilastatin**

**Running Title:** Pharmacokinetic profile of funobactam in renal impairment

**Authors**

Keith A. Rodvold^a^, Grigor Mamikonyan^b^, Yu Wang^c^, Anastasia McRoberts^d^, Dyal Garg^e^, Richard Preston^f^, Thomas Marabury^g^, Meijie Le^c^, Jianguo Li^h^

^a^ University of Illinois Chicago, Chicago, Illinois, USA

^b^ Clinartis LLC, Hollywood, Florda, USA

^c^ Evopoint Biosciences Co., Ltd., Suzhou, China

^d^ Pharmaron (Germantown) Lab Services Inc., Germantown, MD, USA

^e^ Clinical Research Services, Inc., Boynton Beach, FL, USA

^f^ Division of Clinical Pharmacology, University of Miami, Miami, FL, USA

^g^ Orlando Clinical Research Center, Orlando, FL, USA

^h^ Evopoint Biosciences USA, Inc., MA, USA

Table S1	The geometric mean ratios and 90% confidence intervals of pharmacokinetic parameters of funobactam in participants with mild, moderate, and severe renal impairment, and ESRD requiring hemodialysis

|  |  |  | Renal Impairment | | | |
| --- | --- | --- | --- | --- | --- | --- |
| Parameter (units) | Statistic | Normal Renal Function (N=8) | Mild (N=8) | Moderate (N=8) | Severe (N=7) | ESRD (N=8) |
| C_max_ (µg/mL) | LS Geo Mean (SE) | 15.1 (1.07) | 16.6 (1.07) | 15.6 (1.07) | 17.0 (1.07) | 12.7 (1.07) |
|  | LS Geo Mean Ratio (90% CI) ^a^ |  | 1.10  (0.90, 1.34) | 1.03 (0.84, 1.26) | 1.12 (0.91, 1.38) | 0.84 (0.69, 1.03) |
| AUC_0-∞_ (µg*h/mL) | LS Geo Mean (SE) | 46.1 (1.08) | 60.7 (1.08) | 103 (1.08) | 165 (1.08) | 442 (1.07) |
|  | LS Geo Mean Ratio (90% CI) ^a^ |  | 1.32 (1.04, 1.66) | 2.25 (1.78, 2.84) | 3.58 (2.81, 4.56) | 9.58 (7.59, 12.10) |
| t_1/2_ (h) | LS Geo Mean (SE) | 2.89 (1.07) | 3.60 (1.07) | 5.83 (1.07) | 10.1 (1.07) | 25.4 (1.07) |
|  | LS Geo Mean Ratio (90% CI) ^a^ |  | 1.25 (1.01, 1.53) | 2.02 (1.64, 2.49) | 3.48 (2.81, 4.32) | 8.80 (7.15, 10.8) |
| CL (L/h) | LS Geo Mean (SE) | 5.43 (1.08) | 4.12 (1.08) | 2.42 (1.08) | 1.51 (1.08) | 0.57 (1.08) |
|  | LS Geo Mean Ratio (90% CI) ^a^ |  | 0.76 (0.60, 0.96) | 0.45 (0.35, 0.56) | 0.28 (0.22, 0.36) | 0.10 (0.08, 0.13) |
| V_SS_ (L) | LS Geo Mean (SE) | 18.8 (1.06) | 17.5 (1.06) | 18.3 (1.06) | 19.9 (1.06) | 21.6 (1.06) |
|  | LS Geo Mean Ratio (90% CI) ^a^ |  | 0.93 (0.78, 1.11) | 0.97 (0.82, 1.16) | 1.06 (0.88, 1.27) | 1.15 (0.96, 1.37) |
| V_Z_ (L) | LS Geo Mean (SE) | 22.6 (1.06) | 21.4 (1.06) | 20.3 (1.06) | 22.0 (1.07) | 20.8 (1.06) |
|  | LS Geo Mean Ratio (90% CI) ^a^ |  | 0.95 (0.78, 1.15) | 0.90 (0.74, 1.09) | 0.97 (0.79, 1.19) | 0.92 (0.75, 1.12) |

Cohort 1, normal renal function; Cohort 2, Mild RI; Cohort 3, Moderate RI; Cohort 4, Severe RI; Cohort 5, ESRD requiring hemodialysis

Abbreviations: RI, renal impairment; ESRD, end stage renal disease requiring hemodialysis (HD); LS, least square; Geo Mean, geometric mean; SE = standard error; CI, confidence interval; C_max_, maximum plasma concentration; AUC_0-∞_, area under the concentration-time curve time 0 to infinity; t_1/2_, elimination half-life; CL, clearance; volume of distribution (V) at steady-state (V_SS_) and during the terminal elimination phase (V_Z_)

^a^ The LS Geo Mean Ratio (90% CI) for each cohort is computed with respect to Cohort 1

The C_max_ and AUC_0-∞_ parameters for severe renal impairment and ESRD requiring hemodialysis were scaled by 2.5 to match the same dose administered for normal renal function and mild and moderate renal impairment before performing the ANOVA analysis

Table S2	Summary of regression analysis of C_max_, AUC_0-∞_, and CL for funobactam and renal function variable

| Dependent Variable | Independent Variable | Coefficient of Correlation | Slope | 95% CI for Slope | p-value |
| --- | --- | --- | --- | --- | --- |
| C_max_ (µg/mL) | eGFR^a^ | 0.10 | 0.01 | -0.02, 0.03 | 0.539 |
|  | CL_CR_^b^ | 0.07 | 0.01 | -0.02, 0.03 | 0.694 |
| AUC_0-∞_ (µg*h/mL) | eGFR^a^ | -0.77 | -3.34 | -4.25, -2.43 | <0.001 |
|  | CL_CR_^b^ | -0.77 | -3.47 | -4.42, -2.53 | <0.001 |
| CL (L/h) | eGFR^a^ | 0.96 | 0.05 | 0.05, 0.06 | <0.001 |
|  | CL_CR_^b^ | 0.97 | 0.05 | 0.05, 0.06 | <0.001 |

Abbreviations: C_max_, maximum plasma concentration; AUC_0-∞_, area under the concentration-time curve time 0 to infinity; CL, clearance; eGFR, estimated glomerulus filtration rate; CL_CR_, estimated creatinine clearance; CI, confidence interval

^a^eGFR, estimated glomerulus filtration rate calculated by the MDRD formula, transformed to unadjusted eGFR, and with units of mL/minute

^b^ CL_CR_, estimated creatine clearance calculated by the Cockcroft-Gault equation and with units of mL/minute

Table S3	The geometric mean ratios and 90% confidence intervals of pharmacokinetic parameters of imipenem in participants with mild, moderate, and severe renal impairment, and ESRD requiring hemodialysis

|  |  |  | Renal Impairment | | | |
| --- | --- | --- | --- | --- | --- | --- |
| Parameter  (units) | Statistic | Normal Renal Function (N=8) | Mild (N=8) | Moderate (N=8) | Severe (N=7) | ESRD (N=8) |
| C_max_ (µg/mL) | LS Geo Mean (SE) | 25.2 (1.07) | 29.7 (1.07) | 30.1 (1.07) | 35.1 (1.08) | 25.6 (1.07) |
|  | LS Geo Mean Ratio (90% CI) ^a^ |  | 1.18 (0.94, 1.47) | 1.19 (0.95, 1.49) | 1.39 (1.10, 1.76) | 1.01 (0.81, 1.27) |
| AUC_0-∞_ (µg*h/mL) | LS Geo Mean (SE) | 47.4 (1.09) | 64.7 (1.09) | 88.1 (1.09) | 125 (1.10) | 128 (1.09) |
|  | LS Geo Mean Ratio (90% CI) ^a^ |  | 1.37 (1.04, 1.79) | 1.86 (1.42, 2.43) | 2.63 (1.99, 3.48) | 2.69 (2.06, 3.52) |
| t_1/2_ (h) | LS Geo Mean (SE) | 1.38 (1.08) | 1.72 (1.08) | 2.10 (1.08) | 2.59 (1.08) | 3.42 (1.08) |
|  | LS Geo Mean Ratio (90% CI) ^a^ |  | 1.25 (0.99, 1.57) | 1.52 (1.21, 1.92) | 1.88 (1.48, 2.38) | 2.47 (1.96, 3.12) |
| CL (L/h) | LS Geo Mean (SE) | 10.6 (1.09) | 7.72 (1.09) | 5.68 (1.09) | 4.01 (1.10) | 3.92 (1.09) |
|  | LS Geo Mean Ratio (90% CI) ^a^ |  | 0.73 (0.56, 0.96) | 0.54 (0.41, 0.70) | 0.38 (0.29, 0.50) | 0.37 (0.28, 0.49) |
| V_SS_ (L) | LS Geo Mean (SE) | 18.7 (1.07) | 17.3 (1.07) | 16.3 (1.07) | 14.1 (1.08) | 19.3 (1.07) |
|  | LS Geo Mean Ratio (90% CI) ^a^ |  | 0.93 (0.74, 1.16) | 0.87 (0.70, 1.09) | 0.76 (0.60, 0.95) | 1.03 (0.83, 1.29) |
| V_Z_ (L) | LS Geo Mean (SE) | 21.1 (1.08) | 19.2 (1.08) | 17.2 (1.08) | 15.0 (1.09) | 19.3 (1.08) |
|  | LS Geo Mean Ratio (90% CI) ^a^ |  | 0.91 (0.72, 1.16) | 0.82 (0.64, 1.04) | 0.71 (0.56, 0.91) | 0.92 (0.72, 1.17) |

Cohort 1, normal renal function; Cohort 2, Mild RI; Cohort 3, Moderate RI; Cohort 4, Severe RI; Cohort 5, ESRD requiring hemodialysis

Abbreviations: RI, renal impairment; ESRD, end stage renal disease requiring hemodialysis (HD); LS, least square; Geo Mean, geometric mean; SE = standard error; CI, confidence interval ; C_max_, maximum plasma concentration; AUC_0-∞_, area under the concentration-time curve time 0 to infinity; t_1/2_, elimination half-life; CL, clearance; volume of distribution (V) at steady-state (V_SS_) and during the terminal elimination phase (V_Z_)

^a^ The LS Geo Mean Ratio (90% CI) for each cohort is computed with respect to Cohort 1

The C_max_ and AUC_0-∞_ parameters for Cohorts 4 and 5 were scaled by 2.5 to match the same dose administered for Cohorts 1 to 3 before performing the ANOVA analysis

Table S4	Summary of regression analysis of C_max_, AUC_0-∞_, and CL for imipenem and renal function variable

| Dependent Variable | Independent Variable | Coefficient of Correlation | Slope | 95% CI for Slope | p-value |
| --- | --- | --- | --- | --- | --- |
| C_max_ (µg/mL) | eGFR^a^ | -0.26 | -0.06 | -0.13, 0.01 | 0.116 |
|  | CL_CR_^b^ | -0.25 | -0.06 | -0.13, 0.02 | 0.129 |
| AUC_0-∞_ (µg*h/mL) | eGFR^a^ | -0.73 | -0.96 | -1.25, -0.66 | <0.001 |
|  | CL_CR_^b^ | -0.71 | -0.96 | -1.28, -0.64 | <0.001 |
| CL (L/h) | eGFR^a^ | 0.93 | 0.07 | 0.06, 0.08 | <0.001 |
|  | CL_CR_^b^ | 0.93 | 0.07 | 0.06, 0.08 | <0.001 |

Abbreviations: C_max_, maximum plasma concentration; AUC_0-∞_, area under the concentration-time curve time 0 to infinity; CL, clearance; eGFR, estimated glomerulus filtration rate; CL_CR_, estimated creatinine clearance; CI, confidence interval

^a^eGFR, estimated glomerulus filtration rate calculated by the MDRD formula, transformed to unadjusted eGFR, and with units of mL/minute

^b^ CL_CR_, estimated creatine clearance calculated by the Cockcroft-Gault equation and with units of mL/minute

Table S5	The geometric mean ratios and 90% confidence intervals of pharmacokinetic parameters of cilastatin in participants with mild, moderate, and severe renal impairment, and ESRD requiring hemodialysis

|  |  |  | Renal Impairment | | | |
| --- | --- | --- | --- | --- | --- | --- |
| Parameter  (units) | Statistic | Normal Renal Function (N=8) | Mild (N=8) | Moderate (N=8) | Severe (N=7) | ESRD (N=8) |
| C_max_ (µg/mL) | LS Geo Mean (SE) | 31.4 (1.07) | 33.6 (1.07) | 35.5 (1.07) | 43.4 (1.08) | 31.5 (1.07) |
|  | LS Geo Mean Ratio (90% CI) ^a^ |  | 1.07 (0.86, 1.33) | 1.13 (0.91, 1.40) | 1.38 (1.10, 1.73) | 1.00 (0.81, 1.25) |
| AUC_0-∞_ (µg*h/mL) | LS Geo Mean (SE) | 51.4 (1.11) | 65.2 (1.11) | 105 (1.11) | 213 (1.12) | 779 (1.11) |
|  | LS Geo Mean Ratio (90% CI) ^a^ |  | 1.27 (0.90, 1.78) | 2.04 (1.46, 2.87) | 4.15 (2.92, 5.90) | 15.15 (10.8, 21.3) |
| t_1/2_ (h) | LS Geo Mean (SE) | 1.31 (1.10) | 1.55 (1.10) | 2.20 (1.10) | 3.85 (1.11) | 18.8 (1.10) |
|  | LS Geo Mean Ratio (90% CI) ^a^ |  | 1.18 (0.86, 1.62) | 1.68 (1.23, 2.31) | 2.94 (2.12, 4.08) | 14.3 (10.5, 19.6) |
| CL (L/h) | LS Geo Mean (SE) | 9.72 (1.11) | 7.67 (1.11) | 4.75 (1.11) | 2.34 (1.12) | 0.64 (1.11) |
|  | LS Geo Mean Ratio (90% CI) ^a^ |  | 0.79 (0.56, 1.11) | 0.49 (0.35, 0.69) | 0.24 (0.17, 0.34) | 0.07 (0.05, 0.09) |
| V_SS_ (L) | LS Geo Mean (SE) | 15.3 (1.07) | 15.6 (1.07) | 14.3 (1.07) | 12.3 (1.08) | 17.4 (1.07) |
|  | LS Geo Mean Ratio (90% CI) ^a^ |  | 1.02 (0.82, 1.28) | 0.94 (0.75, 1.17) | 0.81 (0.64, 1.02) | 1.14 (0.91, 1.42) |
| V_Z_ (L) | LS Geo Mean (SE) | 18.4 (1.07) | 17.2 (1.07) | 15.1 (1.07) | 13.0 (1.08) | 17.4 (1.07) |
|  | LS Geo Mean Ratio (90% CI) ^a^ |  | 0.93 (0.75, 1.17) | 0.82 (0.66, 1.03) | 0.71 (0.56, 0.89) | 0.95 (0.76, 1.18) |

Cohort 1, normal renal function; Cohort 2, Mild RI; Cohort 3, Moderate RI; Cohort 4, Severe RI; Cohort 5, ESRD requiring hemodialysis

Abbreviations: RI, renal impairment; ESRD, end stage renal disease requiring hemodialysis (HD); LS, least square; Geo Mean, geometric mean; SE = standard error; CI, confidence interval ; C_max_, maximum plasma concentration; AUC_0-∞_, area under the concentration-time curve time 0 to infinity; t_1/2_, elimination half-life; CL, clearance; volume of distribution (V) at steady-state (V_SS_) and during the terminal elimination phase (V_Z_)

^a^ The LS Geo Mean Ratio (90% CI) for each cohort is computed with respect to Cohort 1

The C_max_ and AUC_0-∞_ parameters for Cohorts 4 and 5 were scaled by 2.5 to match the same dose administered for Cohorts 1 to 3 before performing the ANOVA analysis

Table S6	Summary of regression analysis of C_max_, AUC_0-∞_, and CL for cilastatin and renal function variable

| Dependent Variable | Independent Variable | Coefficient of Correlation | Slope | 95% CI for Slope | p-value |
| --- | --- | --- | --- | --- | --- |
| C_max_ (µg/mL) | eGFR^a^ | -0.28 | -0.07 | -0.15, 0.01 | 0.087 |
|  | CL_CR_^b^ | -0.26 | -0.07 | -0.15, 0.02 | 0.112 |
| AUC_0-∞_ (µg*h/mL) | eGFR^a^ | -0.69 | -6.16 | -8.29, -4.03 | <0.001 |
|  | CL_CR_^b^ | -0.69 | -6.40 | -8.61, -4.18 | <0.001 |
| CL (L/h) | eGFR^a^ | 0.96 | 0.09 | 0.08, 0.10 | <0.001 |
|  | CL_CR_^b^ | 0.96 | 0.10 | 0.09, 0.11 | <0.001 |

Abbreviations: C_max_, maximum plasma concentration; AUC_0-∞_, area under the concentration-time curve time 0 to infinity; CL, clearance; eGFR, estimated glomerulus filtration rate; CL_CR_, estimated creatinine clearance; CI, confidence interval

^a^eGFR, estimated glomerulus filtration rate calculated by the MDRD formula, transformed to unadjusted eGFR, and with units of mL/minute

^b^ CL_CR_, estimated creatine clearance calculated by the Cockcroft-Gault equation and with units of mL/minute
